# Neighbourhood demolition, relocation and health. A qualitative longitudinal study of housing-led urban regeneration in Glasgow, UK

**DOI:** 10.1016/j.healthplace.2015.02.006

**Published:** 2015-05

**Authors:** Matt Egan, Louise Lawson, Ade Kearns, Ellie Conway, Joanne Neary

**Affiliations:** a London School of Hygiene & Tropical Medicine, London, United Kingdom; b Urban Studies, School of Social and Political Sciences, University of Glasgow, Glasgow, United Kingdom; c MRC/CSO Social and Public Health Sciences Unit, University of Glasgow, Glasgow, United Kingdom

**Keywords:** Housing, Regeneration, Relocation, Neighbourhoods, Public health

## Abstract

We conducted a qualitative longitudinal study to explore how adult residents of disadvantaged urban neighbourhoods (Glasgow, UK) experienced neighbourhood demolition and relocation. Data from 23 households was collected in 2011 and 2012. Some participants described moves to new or improved homes in different neighbourhoods as beneficial to their and their families’ wellbeing. Others suggested that longstanding illnesses and problems with the new home and/or neighbourhood led to more negative experiences. Individual-level contextual differences, home and neighbourhood-level factors and variations in intervention implementation influence the experiences of residents involved in relocation programmes.

## Highlights

• Housing-led urban regeneration can improve social determinants of health but can also be socially disruptive.
• We found qualitative evidence of complex experiences during regeneration involving neighbourhood demolition and relocation.
• Individual, household, home and neighbourhood characteristics influence residents’ positive and negative experiences.
• Longstanding illness was a particular barrier to residents benefiting from improved residential environments.

## 1 Background

‘Urban regeneration’ describes the restoration and redevelopment of physical and social environments in urban areas that have experienced economic and environmental decline. It sometimes involves large scale housing clearance, demolition, relocation and home improvement programmes. This paper focuses on a housing-led programme of urban regeneration that includes these dimensions.

Systematic reviews have found that, with the exception of certain forms of housing improvement (notably heating improvement), housing-led urban regeneration is poorly evidenced in terms of impacts on health and its social determinants (Gibson et al., 2011b; Jacobs et al., 2010; Thomson et al., 2006b). Nonetheless, there is a commonly stated public health policy expectation that improvements to residential environment (homes and neighbourhoods) can help achieve public health goals of illness prevention and reductions in social inequalities in health by improving determinants of health for disadvantaged populations (Commission on Social Determinants of Health, 2008; Marmot et al., 2010). We have conducted a qualitative longitudinal study to explore how residents of disadvantaged urban neighbourhoods (Glasgow, UK) differentially experience housing clearance, demolition and relocation.

## 2 Theorising pathways from relocation to health improvement

Subsidised relocation to improved or newly built housing is assumed to help disadvantaged residents overcome material (including financial) barriers to obtaining better quality accommodation (Benzeval et al., 2014). Better housing can mean improvements to affordable warmth, ventilation and exposure to damp (Basham et al., 2004; Caldwell et al., 2001; Ellaway et al., 2000; Gibson et al., 2011b; Harrington et al., 2005; Rugkåsa et al., 2004, Thomson et al., 2013). Such improvements are theorised to reduce health risks from injury, biological agents and chemical pollutants (Jacobs et al., 2010; Thomson and Thomas, 2015).

Qualitative research exploring the mechanisms by which moves into better quality homes impacts on exposed populations have suggested that increased indoor and garden space (Bullen et al., 2008; Gibson et al., 2011a), reduced noise (Gibson et al., 2011a) and increased pride and satisfaction (Basham et al., 2004; Bullen et al., 2008; Gibson et al., 2011a; Gilbertson et al., 2006) may benefit health and wellbeing through psychosocial pathways. Health behaviours may in theory be affected by improved kitchens that encourage more time spent on home cooking, and more space – including garden space – for physical activities (Thomson and Thomas, 2015).

Relocation could also theoretically benefit health and wellbeing if the move leads to sufficient improvements in exposures to neighbourhood-level determinants of health (Benzeval et al., 2014). Such improvements may relate to the quality of local services, the presence of amenities that encourage physical activity and other ‘healthy’ behaviours (Gibson et al., 2011a), reduced exposure to ‘unhealthy’ amenities (e.g. high density alcohol and fast food outlets) and improvements in the social environment (Benzeval et al., 2014). Relocations that provide residents with a perception of enhanced social status may lead to psychosocial health benefits (Kearns and Mason, 2013, Kearns et al., 2013).

Despite the numerous theories that explain how housing-led regeneration might improve health, evaluations have tended to provide equivocal results (Jacobs et al., 2010; Thomson et al., 2006a, 2013). An evaluation of a USA housing voucher scheme found that moving from a high-poverty to lower-poverty neighbourhoods improved adult physical and mental health and well-being, despite not affecting economic self-sufficiency (Ludwig et al., 2012). A systematic review of housing improvement and relocation found outcomes varied by study and intervention and were often modest (Thomson et al., 2013). Fullilove (2004) has emphasised the negative social impacts of neighbourhood demolition on local communities in the USA. However, the quantitative arm of the current study found that residents living in neighbourhoods undergoing demolition experienced little or no short term effects on self-rated physical and mental health (Egan et al., 2013).

In the only published qualitative longitudinal study of housing relocations that we know of, the personal circumstances of four households were described in detail to demonstrate how multiple factors and events in each householders’ life interacted to produce widely varying experiences of what was ostensibly the same intervention (Goetz, 2013). This suggests that a simple model of environmental health impacts on exposed populations could not do justice to the complex interactions between individuals, communities and their environment over time, a point that has been made in other qualitative research of relocation interventions (Pinder et al., 2009). The findings also echo Hawe et al. (2009)’s depiction of social interventions as disruptions to complex systems with outcomes that are context-dependent, non-linear and unpredictable.

There remains a question as to whether substantial improvements to health can be realistically achieved in deprived urban neighbourhoods without first, or at least concurrently, engaging in what we have termed ‘social regeneration’: i.e. addressing the fundamental characteristics of deprivation (low income, low employment, etc.). We have reported elsewhere our view that housing-related outputs may appear to policy-makers and planners to be more deliverable in disadvantaged areas than tackling the socio-structural causes of inequalities and disadvantage. Hence, ‘social regeneration’ may at times be deprioritised in favour of physical improvements to residential environments (Kearns et al., 2013).

## 3 Aims

The current study focuses on residents at the crucial period of a clearance and demolition programme when relocation to new or improved properties occurred as part of a city-wide housing-led regeneration programme in Glasgow, UK. Our aim was to explore in depth the experiences of residents during this period in order to identify mechanisms by which neighbourhood demolition involving large scale resident clearance and relocation may differentially impact upon health and wellbeing.

## 4 Methods

### 4.1 Study background

The Lived Realities study is a qualitative longitudinal component of a wider research programme called [name removed], evaluating the effects of urban regeneration on residents in disadvantaged neighbourhoods of Glasgow, UK (Egan et al., 2010).

### 4.2 Settings

Three inner-city mass-housing estates undergoing large scale clearance and demolition were selected for the study. Over 90% of homes were socially rented: i.e. homes that are let by public or third sector organisations (e.g. Housing Associations) at below-market rents to people in housing need. The estates were comprised predominantly of high-rise blocks, each met the Scottish Government’s definition of disadvantaged areas (Walsh, 2008), and each contained a mixture of UK-born residents and first generation migrants (mainly asylum-seekers and refugees). In 2011, Areas A, B and C contained approximately 1300, 700, and 800 occupied dwellings respectively.

### 4.3 Intervention

Between 2006 and 2011, over 60% of the homes in each neighbourhood were cleared and either prepared for demolition or actually demolished. Those who still remained in the areas were awaiting relocation: a process that involved interviews with local housing officers, viewing usually up to three social rented properties in other areas for suitability, and receiving a modest relocation payment to help with expenses (Kearns and Darling, 2013). Residents tended to relocate to nearby neighbourhoods in homes that were newly built or had been recently refurbished to meet new national standards. At a future point the original neighbourhoods will be rebuilt but this is not the focus of the current paper, as completion is not due for at least another decade. Here, we focus on residents obliged to relocate from neighbourhoods being demolished.

### 4.4 Data collection

Interviews were conducted with adult householders and/or partners. Participants were recruited via local housing associations, church/community groups, snowballing and the [name of study] survey. The interviews were loosely structured around themes including the participants’ background, everyday activities, home and neighbourhood, wellbeing and aspirations. Twenty-three households participated at Wave 1 (W1). A year later (W2), we re-interviewed participants from 12 of these households (see Table 2). Participants did not all participate in both waves. Due to the staggered and complex nature of the rehousing programme, participants were in different stages of the process and not all were relocated during the course of data collection—further details can be seen in Table 1. Whilst we had originally prioritised family households, we also decided to interview three participants who each lived alone to gain an insight into their experiences of relocation. The participants therefore included a wide range of the kinds of household structures, age groups, nationalities and employment types that occurred in each of these neighbourhoods (see Table 2).

The University of Glasgow’s ethics committee approved the study and its procedures for informed consent, data protection and confidentiality. Digital audio recordings of the interviews were transcribed by a specialist transcription company. Participants received £20 in shopping vouchers to thank them for their time. Each participant was given a pseudonym.

### 4.5 Data analysis procedures

The analytical approach was inductive and ‘bottom-up’, drawing on aspects of thematic analysis and phenomenological analysis (Benner, 1985). The analysis aimed to develop insightful interpretation anchored in the participants’ accounts (Smith et al., 2009). Transcribed interviews were analysed by two researchers using a coding framework developed jointly. Our analysis focused on residents perceptions of health and the residential environment prior to moving. Then using data from those participants who had relocated (at either wave), we focus on potential pathways from relocation to health and wellbeing. At this latter stage we also considered the experiences of participants still waiting to be rehoused by W2, as this prolonged wait for resettlement was an important aspect of how some residents experienced the intervention.

#### 4.5.1 Findings

The findings are split into two main sections in line with the analysis strategy reported above First, we examine perceptions of health, housing and neighbourhoods prior to relocation. Second, we explore these issues after relocation and identify potential pathways from relocation to health and wellbeing. We also include experiences of delayed relocation in this second section.

Therefore, we begin by describing residents’ perceptions and experiences of their original residential environments. The main themes in this section are the diverse range of health problems experienced by residents; and perceptions that both homes and neighbourhoods can influence health and wellbeing (although they are not seen as the only influences).

### 4.6 Perceptions of health, housing and neighbourhoods before relocation

#### 4.6.1 Experiences of morbidity

In keeping with the high prevalences of morbidity known to exist in these neighbourhoods (Walsh, 2008), most of the participants stated that either they or another household member had multiple and/or longstanding health problems. Physical illnesses described included asthma, eczema, sciatica, kidney problems affecting the immune system, kidney stones, pneumonia, AIDS, ulcers, diabetes, brain malformation, back pain, amputations and arthritis. Psychological issues included anxiety, depression, self-harm and violent conduct. Some participants identified themselves as recovering alcohol, heroin or amphetamine misusers and several smoked. For some, longstanding illness posed barriers to employment and mobility, obliging participants to spend more time exposed to home and neighbourhood environments.

Participants gave wide ranging explanations for health problems that included early life disadvantage, genetic factors, behaviours and different types of environmental exposures. However, many also made clear their view that health problems had been caused or, more typically, exacerbated by current problems with their homes and neighbourhoods. We focus on these below.

#### 4.6.2 Damp and cold homes

Participants frequently characterised their homes as damp and cold, and there was a common belief that these problems affected family health and wellbeing. Sue claimed that one of her sons moved out of her damp flat in an attempt to alleviate his asthma. Rachel and Keith believed their damp home had adversely affected the health of all three of their children:

*Rachael*: Every one of them has not been well.

*Keith*: She [his daughter] gets asthma you see and that’s why she’s [got her bed] away from the window…because obviously these windows are rubbish.

Heating systems that used pay-as-you-go cards (‘powercards’) were considered a particular barrier to affordable warmth, as powercards were understood to be more expensive than standard billing (as reported by Barbara, Paul and Aisha). Aisha believed that the cost of heating her flat was a barrier to managing her diabetes:

*Aisha*: Trying to say to the social security [welfare provider], because I’m diabetic, I need baths and heaters quite constantly. And the bill—I pay the powercard and I go through a lot.

#### 4.6.3 Inadequately sized homes

Some participants reported that lack of space adversely affected their mental health. Morag was dealing with several major life-issues including long term unemployment, HIV and the methadone programme, but also believed that her single-room apartment contributed to her depression at W1. She stated that “if I got a better house I think that would help me”. Some homes were overcrowded: Nada’s three bedroom flat accommodated a family of seven; Keith and Rachel’s two bedroom flat accommodated five. Furthermore, Anne, Moira, Rachel, Keith and Sue all described how damp reduced useable living space, as furniture had to be moved in from walls and, in the worst cases, entire rooms abandoned. Livingroom sofas were used as beds in many of the participating W1 households, which could strain family relationships. Carol, a lone parent who suffered from depression, described her livingroom on a typical evening:

*Carol:* The oldest [child] is normally into her laptop with her earphones on and the youngest is sitting with her [games console]…and I’ll say ‘I want to get my bed out’. And then they’ll end up getting stuck on one couch then they’ll start arguing—I’m not getting any peace… it has been driving me nuts.

The above example illustrates how problems with the home physical environment can affect health and wellbeing through multiple mechanisms. A lack of space may impact on health directly as residents find their ability to rest and sleep limited by a physical home environment that exposes them to noise and disturbance. Negative impacts may also follow social and psychosocial pathways, as crowded conditions lead to family arguments and remove householders’ sense of privacy and control.

#### 4.6.4 Home shame

A potential psychosocial impact of poor quality housing stems from the sense of shame and embarrassment that many participants (Jon, Morag, Alison, Layan, Sue, Carol, Paul, and Aisha) expressed about their living conditions. Shame can be a barrier to social connectivity and support (Dean and Hastings, 2000), and participants claimed it deterred both themselves and their children from inviting friends and family round to visit. Hence Alison hoped that relocation would bring her “peace, contentment, feeling safe in your own house, a house that you can live in and that you’re not ashamed to bring people into—because this house is a mess”.

#### 4.6.5 Neighbourhood physical environment

Given the large scale of neighbourhood demolition, surprisingly few participants drew attention to the ongoing destruction of their neighbourhoods. Alison was an exception, as she associated the intervention with perceptions of lawlessness and crime. After describing how local buildings were cleared and demolished she added “everything has totally changed. I say, at times, it’s like The Bronx, here. It’s like the Bronx. Every year, there’s at least two murders.”

Physical and psychosocial factors were presented in combination as participants talked about public spaces that felt unsafe. Participants complained that their children’s opportunities for outdoor activity were restricted by vandalised parks and perceptions of danger (Layan, Paul). Common areas of high-rised flats, such as stairways and lifts, were criticised for encouraging and placing participants in close proximity to a range of anti-social behaviours. These included intoxicated and intimidating neighbours (Sami), urine (Maya, Moira), smoking, alcohol, drug use and discarded needles (Sami, Keith, Carol). Paul, a married father who relocated to a newly built house prior to W1, talked of how his son’s school friends refused to come round to the old flat because “a lot of them were frightened of the lifts.” When Morag’s mother visited they would arrange to meet in a local cafe because “she doesn’t like the lifts…she hates it.”

#### 4.6.6 Neighbourhood social networks and community

Some participants did worry about how their own imminent rehousing might lead to separation from friends. Barbara, who lived alone and was being treated for HIV, said that “emotionally, I would die inside” if she was separated from a neighbour she was close to. Other participants avoided their neighbours and some described their loneliness. The local drinking culture played a role in Aisha’s isolation: her diabetes onset obliged her to avoid alcohol but she found it difficult to socialise whilst staying clear of people and environments that encouraged her drinking:

*Aisha*: Before [being diagnosed diabetic], I used to love the music, go to the dancing, have a good drink. Because I’m diabetic I don’t really drink now, it’s a problem…There used to be a lot of music. But now I put music on, I just get a wee bit greetful [little bit tearful]…I thought maybe it’s me, missing my friends, missing the good times.

Asylum seekers and refugees recounted incidents of UK-born neighbours engaging in racially motivated verbal (Nadia, Nada, Sami) and physical (Nadia, Layan) abuse. However, a more positive narrative can also be found describing a growing sense of pride and attachment towards their neighbourhoods and neighbours (Nada, Layan, Ula, Moira). Ula insisted that she and her family were happy to live in Area A:

*Ula*: I’m involved in [the local] community, I’m enjoying my life here. Is not stressful. I love [Area A].

In contrast, it was difficult to find examples of UK-born participants declaring a strong attachment to their neighbourhood. What praise there was tended to be expressed with reservations. For example, Jackie claimed to like Area A but emphasised how she kept some distance from the community: “I like [Area A] for the reason that, I just keep myself to myself.”

### 4.7 Potential pathways to health and wellbeing

We turn now to the experiences of residents who relocated at W1 and W2 and residents still waiting to relocate by W2. We have grouped these experiences into three broad themes: (i) residents experiencing little change or improvement to either their residential environment or their health and wellbeing; (ii) residents experiencing perceived improvements to residential environments but not to health and wellbeing; and (iii) residents experiencing perceived improvements to residential environments, health and wellbeing.

**Fig. 1:**
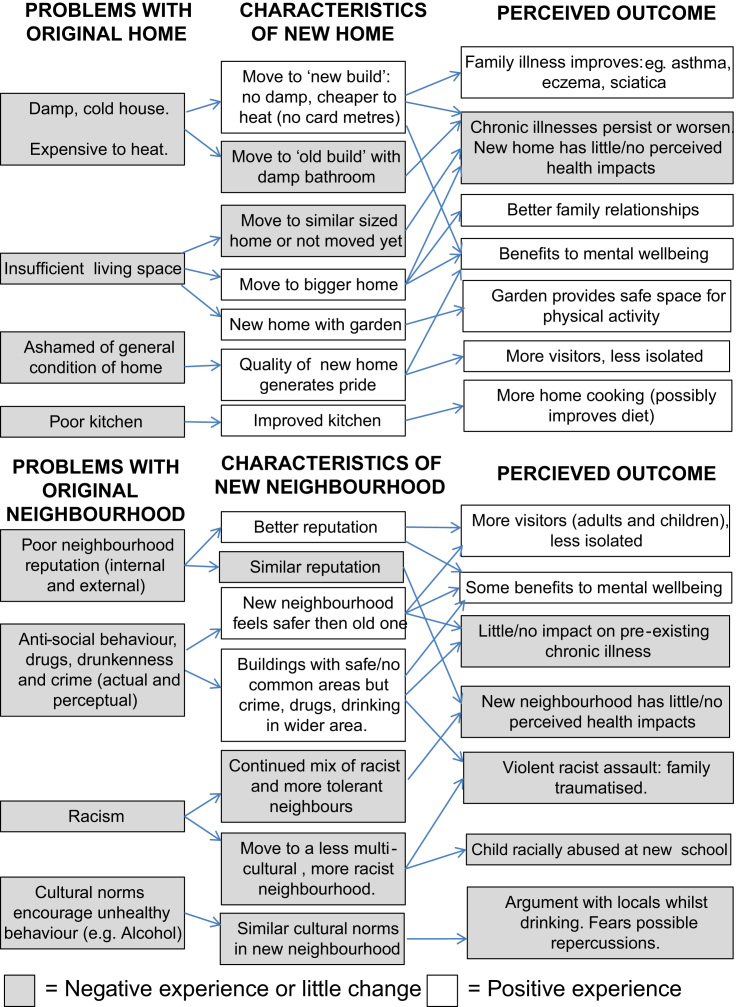
Pathways to perceived health and wellbeing.

Although we have used this three theme structure to organise our analysis, participants’ individual experiences were much more varied and complex than this simple categorisation might suggest. Furthermore, the three themes are not mutually exclusive in that residents could perceive improvements in one aspect of their environment or health but not another. We therefore include Fig. 1 to emphasise the complexity and variety of pathways suggested by participant accounts, as a kind of counterweight to our more simplified categorisation of findings in the themes discussed below.

#### 4.7.1 Theme 1: No perceived improvements

Barbara and Nada had yet to relocate by W2. Barbara believed that remaining in Area A prolonged her unhealthy exposure to cold and damp, but her social support network continued to function and helped her stay positive. Nada assumed that remaining in her damp flat was a potential health risk but nonetheless said at both waves that she and her household enjoyed good health.

Other participants did relocate but continued to experience problems similar to those identified prior to their moves, albeit in a new context. Carol had moved from a high-rise flat with a major damp problem, to a cottage flat with a more localised damp problem. The move had not alleviated a back problem which she attributed to sleeping on a sofa in the old flat to avoid her damp bedroom. However, Carol said at W2 that she was still in the habit of sleeping on her sofa despite now having her own bedroom and bed. Ula had moved from a multi-cultural neighbourhood (albeit one where racism was still evident) to a less multi-cultural area where she and a friend subsequently suffered a severe racist assault. Contrasting with her optimistic W1 interview, Ula reported at W2 that the assault had “destroyed every happiness” for herself and her family, and made them wish they could return to Area A.

*Ula*: I didn’t feel really lonely, but now when I came here it’s just stressful and I feel like I want to go back.

From the choice of homes offered to Aisha, she had selected a reconditioned flat situated above a pub and was still drinking despite her diabetes. A recent argument with locals at the pub left her feeling anxious about reprisals.

*Aisha*: But I feel scared to go back to that pub…I think there’s people up here that would want to hit you.

#### 4.7.2 Theme 2: Perceived improvement in environment but not health

At W2 some of the participants described experiencing environmental improvements that were not accompanied by perceptions of improved health. For example, Maya’s asthma continued to cause her problems after moving to a good quality (in her opinion) new build house. Her son also began experiencing problems:

*Maya*: Yeah, my boy wasn’t usually sick when we were in [her previous neighbourhood] but recently he’s started…He started itching the eyes and coughing and his nose is blocked. And it started again this year too, his chest was dry so they had to give him inhaler as well.

Some participants suggest that their physical and psychosocial home environment had improved, but the improvements were insufficient to alleviate longstanding anxiety and depression. Having moved to a new build, Sue was still too depressed to socialise. Morag believed her new flat made her feel better for a while (“at first I was all buzzing about”) but her social circumstances and chronic physical health problems were not radically changed and her depression returned.

#### 4.7.3 Theme 3: Perceived improvements to environments and health

Some participants believed that the relocation benefited their health, wellbeing and associated behaviours. Heather and Maya suggested that better equipped kitchens encouraged more home cooking of better quality. Heather said “maybe, the quality of the food’s better because we’ve got a better cooker,” although details of the dietary improvement were not given. They, along with Sue and Layan, also depicted their new gardens as spaces for physical activity such as gardening work or children’s play. Paul believed that moving into a damp-free house that was relatively cheap to heat led to improvements in childhood asthma and eczema.

*Paul*: Living in this house, it’s a lot better. It’s healthier, my kids are not ill. That’s the worst thing in the [previous] flats.

Layan made similar comments about her son’s asthma and her husband’s sciatica improving after her move to a newly built house.

The social and psychosocial benefits of relocation were also alluded to by some participants. Maya stated that moving to a larger home improved family relationships and contributed to the family’s overall sense of happiness.

*Maya*: as you can see there’s a lot of space. Children would have been fighting for space [in the previous flat]… That has changed completely, the bond is more strong because we all sit together and talk to each other.

Several participants commented on how their children were happier now that they lived in neighbourhoods nearer to friends, and in homes that friends could visit without feeling unsafe (Paul, Carol, Layan). The social impacts of relocation were less clear for migrants, who often seemed to have the greatest level of social investment in the original community. Ula, Layan and Maya made regular trips back to their original neighbourhoods to visit friends, attend religious services and engage in voluntary work. Maya reported that her daughter had returned to her previous school after experiencing racially motivated bullying in a school nearer to her new home. Nonetheless, Maya believed that once the school problem was resolved her children were “happier” after the move.

## 5 Discussion

We have used qualitative methods to explore residents’ accounts of their experience of housing, relocation and health. We have identified a variety of experiences and outlined some of the key pathways that appear to underpin those experiences. Residents frequently believed that their residential environment caused or at least exacerbated illness. Some residents also hoped that relocation to better housing would benefit health and wellbeing. Those that did relocate reported a mixture of experiences including benefits, little change and further problems. The various mechanisms for change we identified include changes affecting household damp and affordable warmth, space at home, pride in residents’ homes and neighbourhoods, and safety concerns associated with certain types of public space. These identified mechanisms corroborate findings from other studies, as indicated in Thomson and Thomas (2015)’s recently published synthesis of theories of change associated with housing and relocation.

Our findings present positive and negative narratives of living through a major demolition and relocation programme. This mixture of experiences fits with the previously published evidence base, summarised in the findings of various systematic reviews (Gibson et al., 2011b). For example reviews of housing improvement and relocation (Thomson et al., 2013) and reviews of other forms of housing-led regeneration (Jacobs et al., 2010; Thomson et al., 2006b) highlight positive, inconclusive and adverse effects across a range of interventions and outcomes. However, many of the quantitative findings identified in these reviews tend to find small changes across a limited number of outcomes (Thomson et al., 2013). The qualitative work of Goetz (2013) parallels our own findings by emphasising how residents receiving the same housing intervention can have radically different experiences involving a wide range of outcomes—in contrast to the *no effects* or *small effects* conclusions that we would argue typify many quantitative studies.

### 5.1 Implications for research

Our findings further underline the need for evaluations of complex interventions to capture a range of positive and negative experiences. Otherwise, evaluations risk under-reporting impacts. For example, a methodologically robust quantitative study may accurately identify modest (or no) changes in a specific mean outcome between two time points but fail to explore whether the intervention has had strongly polarised effects across a much wider variety of outcomes. Such studies may well be factually correct, in the broadest sense, but yet miss more important points about the different ways that people’s lives and wellbeing have been affected.

### 5.2 Implications for practice

Our findings suggest specific mechanisms by which relocation may improve health. However they also suggest a need for public health policy-makers to be clearer about how preventative health care strategies can be realised in the context improving social determinants of health in disadvantaged neighbourhoods with high rates of morbidity. In such populations, the term ‘prevention’ is applied somewhat inappropriately to large numbers of people who are already ill (Commission on Social Determinants of Health, 2008; Marmot et al., 2010).

Relocation may be more effective if treated as a critical moment by specialist health agencies to ensure that chronically ill residents’ therapeutic regimes are enhanced rather than disrupted, and that positive feelings associated with the move are built upon to encourage health enhancing lifestyle and behavioural changes. Pre-improvement and pre-relocation discussions with residents about their lives and health may assist service deliverers to enable residents to get the maximum benefit out of residential change and help avoid relocations to inappropriate properties and locations. It should be noted that relocation counselling is a stronger feature of programmes in other countries than it is in the UK (Varady and Kleinhans, 2013).

Our findings also highlight the importance, but also the inadequacy and difficulty of achieving what we have termed ‘social regeneration’: i.e. community level improvements to social and psychosocial environments as well as the promotion of healthier cultural norms and behaviours. However, social and physical environments are not exclusive of one another: a strong theme across the study related to perceived problems of safety and anti-social behaviour within specific physical spaces such as lifts, stairs and other common areas of high rise flats. Relocating to dwellings with less or no internal common areas (and fewer people who use them) seems to remove a source of fear and isolation. Interestingly, this finding echoes, or rather amplifies, findings from a very early study of high-rise living in Glasgow in the 1960s (Jephcott and Robinson, 1971, p. 55).

### 5.3 Strengths and Limitations

This qualitative component of the [name of study] study aims to explore pathways and mechanisms by which regeneration affects different residents, and to consider unintended consequences (whilst the quantitative component measures effects). This kind of mixed methods approach is increasingly accepted as a sensible and necessary way of evaluating complexity (Craig et al., 2011) but it leads to a particular tension: qualitative methods are used to explore issues relating to causality despite wide acceptance of the view that qualitative studies cannot be used to attribute or measure generalisable causal effects. Furthermore, residents’ opinions are subjective and represent a different kind of perspective to that offered by a medically trained researcher, housing officer or health professional. In this study we have used residents’ accounts to hypothesise multiple causal pathways; we have explored residents’ perceptions of their environment and health, including perceptions of how one may affect the other; but we have not attempted to quantify effect sizes or suggest an overall direction of attributable intervention effect(s). The principles underpinning our approach can also be found in previous qualitative research that explore mechanisms by which residential environments affect health (Mehdipanah et al., 2013; Coulson et al., 2011; Warr et al., 2007; Day, 2008).

In terms of generalisablity, the extent to which the findings are applicable to other populations and settings is limited by the purposive and pragmatic sampling of participants and neighbourhoods. However, we know that many places in the UK have high-rise social housing estates and ‘a high proportion of these have technical or social problems’ (Towers, 2000, p. 208, see also Crawford and Walsh, 2010).

## 6 Conclusion

This study highlights the importance of ensuring that regeneration outputs represent genuine improvements compared to previous residential environments. It also highlights the need for broad regeneration strategies that focus on strengthening community cohesion and socio-structural causes of disadvantage as well as simply improving homes. Disadvantaged residents often have illnesses that are not primarily caused by their current residential environment and it can be unrealistic to expect modifications in that environment to influence those health problems substantially. Perhaps this helps explain why residents’ accounts suggest that improvements in specific social determinants of health can at times fail to result in improved health and/or wellbeing. When evaluating the effects of complex social interventions, researchers need to consider the likelihood of wide-ranging and unpredictable impacts. Public health researchers and policy-makers should also consider how the term ‘prevention’ applies to disadvantaged populations already characterised by high levels of morbidity.

## Figures and Tables

**Table 1:** Participants housing status and participation at Waves 1 and 2.

**Pseudonyms**	**Wave 1 (2011) relocation status**	**Traced to wave 2 (2012)?**	**Wave 2 relocation status**
Nada	Original neighbourhood	Yes	Original neighbourhood
Barbara	Original neighbourhood	Yes	Original neighbourhood
Sue	Original neighbourhood	Yes	Relocated
Ula	Original neighbourhood	Yes	Relocated
Layan	Original neighbourhood	Yes	Relocated
Jackie	Original neighbourhood	Yes	Relocated
Aisha	Original neighbourhood	Yes	Relocated
Harry	Original neighbourhood	Yes	Relocated
Morag	Original neighbourhood	Yes	Relocated
Carol	Original neighbourhood	Yes	Relocated
Ali	Original neighbourhood	Unavailable^⁎^	–
Jon	Original neighbourhood	Unable to contact	–
Rachel & Keith	Original neighbourhood	Unable to contact	–
Sami	Original neighbourhood	Unable to contact	–
Alison	Original neighbourhood	Unable to contact	–
May/Dave	Original neighbourhood	Withdrew	–
Moira	Original neighbourhood	Withdrew	–
Maya	[Process of] relocating	Yes	Relocated
Heather & Paul	Relocated	Yes	Relocated (i.e. same address as wave 1)
Lesley	Relocated	Unavailable^⁎^	–
Nadia	Relocated	Unable to contact	–
Basra	Relocated	Withdrew	–
Lynda	Relocated	Withdrew	–
23		12	

*Note*: ‘Original neighbourhood’ refers to the neighbourhoods that were being cleared for demolition. At wave 1, participants either resided in such a neighbourhood, or they had relocated immediately before or during the Wave 1 fieldwork period. ‘Unavailable’ refers to participants who, on contact, said they were not available to participate at Wave 2 but could be available in future should we opt for further follow-up.

**Table 2:** Participants’ household structure and key personal characteristics.

**Pseudonym and wave (W) interviewed**	**Personal characteristics**	**Country of birth**	**Others in household**
Nada (W1,W2)	Female. Voluntary work.	Lebanon	Husband, 5 children.
Barbara (W1, W2)	Female. Unemployed.	UK	None
Sue (W1, W2)	Female. Unemployed.	UK	2 Children.
Ula (W1, W2)	Female. Voluntary work.	Sudan	Husband, 3 children.
Layan (W1, W2)	Female. Voluntary work.	Palestine	Husband, 3 children.
Jackie (W1, W2)	Female. Employed.	UK	3 Children.
Aisha (W1, W2)	Female. Unemployed.	UK	1 Child.
Harry (W1, W2)	Male. Unemployed.	UK	1 Child.
Morag (W1, W2)	Female. Unemployed.	UK	None
Carol (W1, W2)	Female. Employed.	UK	2 Children.
Ali (W1)	Male. Employed, off sick.	Iraq	Wife, baby
Jon (W1)	Male. Unemployed.	Kenya	None
Rachael/Keith (W1)	Female/Male. Unemployed.	UK	3 Children.
Sami (W1)	Male. Employed.	Bangladesh	Wife, baby
Alison (W1)	Female. Unemployed.	UK	2 Children.
May/Dave (W1)	Female/Male. Unemployed.	UK	1 Child.
Moira (W1)	Female. Voluntary work.	UK	1 Grandchild.
Maya (W1, W2)	Female. Employed.	Ghana	Husband, 4 children.
Heather/Paul (W1,W2)	Female/ male. Employed.	UK	2 Children.
Lesley (W1)	Female. Unemployed.	UK	2 Grandchildren.
Nadia (W1)	Female. Employed.	Ivory Coast	Husband, 2 children.
Basra (W1)	Female. Unemployed.	Somalia	Husband, 2 children.
Lynda (W1)	Female. Employed.	UK	1 Child.

*Note*: Some unemployed residents considered it important to emphasise their status as engaged in unpaid work (e.g. charities, religious and self-help groups). Hence, in the table above we use the term ‘voluntary work’ to signify a participant who is not in paid employment but who volunteers.

## References

[bib1] Basham M., Shaw S., Barton A., Torbay H. (2004). Central Heating: Uncovering the Impact on Social Relationships in Household Management.

[bib2] Benner P. (1985). Quality of life: a phenomenological perspective on explanation, prediction, and understanding in nursing science. Adv. Nurs. Sci..

[bib500] Benzeval M., Bond L., Campbell M., Egan M., Lorenc T., Petticrew M., Popham F. (2014). How does money influence health?.

[bib5] Bullen C., Kearns R., Clinton J., Laing P., Mahony F., McDuff I. (2008). Bringing health home: householder and provider perspectives on the healthy housing programme in Auckland, New Zealand. Soc. Sci. Med..

[bib6] Caldwell J., McGowan S., McPhail J., McRae C., Morris G., Murray K. (2001). Glasgow Warm Homes Study: Final Report.

[bib7] Commission on Social Determinants of Health (2008). Closing the Gap in a Generation: Health Equity Through Action on the Social Determinants of Health. Final Report of the Commission on Social Determinants of Health.

[bib8] Coulson J.C., Fox K.R, Lawlor D., Trayers T. (2011). Residents’ diverse perspectives of the impact of neighbourhood renewal on quality of life and physical activity engagement: improvements but unresolved issues. Health Place.

[bib9] Craig P., Cooper C., Gunnell D., Haw S., Lawson K., Macintyre S., Ogilvie D., Petticrew M., Reeves B., Sutton M., Thompson S. (2011). Using Natural Experiments to Evaluate Population Health Interventions.

[bib10] Crawford,F., Walsh,D. (2010) The Wider Relevance of [name of study] to Other Urban Areas in Scotland. Glasgow: [name of study].

[bib11] Day R. (2008). Local environments and older people’s health: dimensions from a comparative study in Scotland. Health Place.

[bib12] Dean J., Hastings A. (2000). Challenging Images: Housing Estates, Stigma and Regeneration.

[bib501] Egan M., Katikireddi S.V., Kearns A., Tannahill C., Kalacs M., Bond L. (2013). Health effects of neighborhood demolition and housing improvement: a prospective controlled study of 2 natural experiments in urban renewal. American Journal of Public Health.

[bib800] Egan M., Kearns A., Mason P., Tannahill C., Bond L., Coyle J., Beck S., Crawford F., Hanlon P., Lawson L., McLean J., Pettigrew M., Sautkina E., Thomson H., Walsh D. (2010). Protocol for a mixed methods study investigating the impact of investment in housing, regeneration and neighbourhood renewal on the health and wellbeing of residents: the GoWell programme. BMC Medical Research Methodology.

[bib17] Ellaway A., Macintyre S., Fairley A. (2000). Mums on Prozac, kids on inhalers: the need for research on the potential for improving health through housing interventions. Health Bull..

[bib18] Fullilove M.T. (2004). Root Shock, How Tearing Up City Neighhorhoods Hurts America, and What We Can Do About It.

[bib19] Gibson M., Thomson H., Kearns A., Petticrew M. (2011). Understanding the psychosocial impacts of housing type: qualitative evidence from a housing and regeneration intervention. Hous. Stud..

[bib20] Gibson M., Petticrew M., Bambra C., Sowden A.J., Wright K.E., Whitehead M (2011). Housing and health inequalities: a synthesis of systematic reviews of interventions aimed at different pathways linking housing and health. Health Place.

[bib21] Gilbertson J., Stevens M., Stiell B., Thorogood N. (2006). Home is where the hearth is: grant recipients’ views of England’s home energy efficiency scheme (Warm Front). Soc. Sci. Med..

[bib22] Goetz E.G. (2013). Too good to be true? The variable and contingent benefits of displacement and relocation among low-income public housing residents. Hous. Stud..

[bib24] Harrington B., Heyman B., Heyman A., Merleau-Ponty N., Ritchie N., Stockton H. (2005). Keeping warm and staying well: findings from the qualitative arm of the Warm Homes Project. Health Soc. Care Community.

[bib25] Hawe P., Shiell A., Riley T. (2009). Theorising Interventions as Events in Systems. Am. J. Community Psychol..

[bib27] Jacobs D.E., Brown M.J., Baeder A., Sucosky M.S., Margolis S., Hershovitz J. (2010). A systematic review of housing interventions and health: introduction, methods, and summary findings. J. Public Health Manage. Pract..

[bib28] Jephcott P., Robinson H. (1971). Homes in High Flats: Some of the Human Problems Involved in Multi-Storey Housing.

[bib503] Kearns, A., Darling, L, Giving the ‘all clear’: housing staff experience of the rehousing process in TransformationalRegeneration Areas, 2013, GoWell; Glasgow

[bib504] Kearns A., Mason P. (2013). Defining and measuring displacement: is relocation from restructured neighbourhoods always unwelcome and disruptive?. Housing Studies.

[bib505] Kearns A., Whitley E., Bond L., Egan M., Tannahill C. (2013). The psychosocial pathway to mental well-being at the local level: investigating the effects of perceived relative position in a deprived area context. Journal of Epidemiology & Community Health.

[bib31] Ludwig J., Duncan G., Gennetian L., Katz L., Kessler R., Kling J. (2012). Neighborhood effects on the long-term well-being of low-income adults. Science.

[bib32] Marmot M., Allen J., Goldblatt P., Boyce T., McNeish D., Grady M. (2010). Fair Society, Healthy Lives: The Marmot Review—Strategic Review of Health Inequalities in England post-2010.

[bib33] Mehdipanah R., Malmusi D., Muntaner C., Borrell C. (2013). An evaluation of an urban renewal program and its effecs on neighbourhood residents’ overall wellbeing using concept mapping. Health Place.

[bib35] Pinder R., Kessel A., Green J., Grundy C (2009). Exploring perceptions of health and the environment: a qualitative study of Thames Chase Community Forest. Health Place.

[bib37] Rugkåsa J., Shortt N., Boydell L. (2004). Engaging Communities: An Evaluation of a Community Development Model for Tackling Rural Fuel Poverty.

[bib38] Smith J.A., Flowers P., Larkin M. (2009). Interpretative Phenomenological Analysis: Theory*,* Method and Research.

[bib43] Thomson H., Thomas S. (2015). Developing empirically supported theories of change for housing investment and health. Soc. Sci. Med..

[bib39] Thomson H., Atkinson R., Petticrew M., Kearns A. (2006). Do urban regeneration programmes improve public health and reduce health inequalities: a synthesis of the evidence from UK policy and practice (1980–2004). J. Epidemiol. Commmunity Health.

[bib40] Thomson H., Atkinson R., Petticrew M., Kearns A. (2006). Do urban regeneration programmes improve public health and reduce health inequalities? A synthesis of the evidence from UK policy and practice (1980–2004). J. Epidemiol. Commmunity Health.

[bib42] Thomson, H., Thomas, S., Sellstrom, E., Petticrew, M. (2013). Housing Improvements for Health and Associated Socio-economic Outcomes. Cochrane Database of Systematic Reviews, 2:Art. No.: CD008657 doi:10.1002/14651858.CD008657.pub2. 10.1002/14651858.CD008657.pub2 PMC12551615 23450585

[bib44] Towers G. (2000). Shelter is Not Enough: Transforming Multi-Storey Housing.

[bib45] Varady D., Kleinhans R. (2013). Relocation counselling and supportive services as tools to prevent negative spillover effects: a review. Hous. Stud..

[bib46] Walsh, D. 2008. Health and Wellbeing in Glasgow and the [name of study] Areas—Deprivation Based Analyses. Glasgow.

[bib47] Warr D.J., Tacticos T., Kelaher M., Klein H. (2007). ׳Money, stress, jobs’: residents’ perceptions of health-impairing factors in ‘poor’ neighbourhoods. Health Place.

